# Supplementary material for: Wing bone laminarity is not an adaptation for torsional resistance in bats
Source: PeerJ. 2015 Mar 5;3:e823. doi: 10.7717/peerj.823 (PMC4359045; doi:10.7717/peerj.823)
Supplement: Table S1 [file peerj-03-823-s001.docx]

Supplementary Table 1 **Bone embedding protocol.**

| **Process** | **Fluid** | **Time** |
| --- | --- | --- |
| Fixation | 10% neutral buffered formalin | Under vacuum, 48 h with one change after 24 h |
| Dehydration A | 70% ethanol | Under vacuum, 48 h with one change after 24 h |
| Dehydration B | 85% ethanol | Under vacuum, 48 h with one change after 24 h |
| Dehydration C | 100% ethanol | Under vacuum, 24 h |
| Clearing | Histo-Clear | Under vacuum, 48 h with one change after 24 h |
| Infiltration A | Resin with 0 g catalyst per 100 mL of resin | Under vacuum, 48 h with one change after 24 h |
| Infiltration B | Resin with 1.4 g catalyst per 100 mL of resin | Under vacuum, 48 h with one change after 24 h |
| Embedding | Resin with 3.5 g catalyst per 100 mL of resin | 48 h in bead bath at 32 °C |
