# Supplementary material for: Wing bone laminarity is not an adaptation for torsional resistance in bats
Source: PeerJ. 2015 Mar 5;3:e823. doi: 10.7717/peerj.823 (PMC4359045; doi:10.7717/peerj.823)
Supplement: Table S2 — Abbreviations: CMNH, Carnegie Museum of Natural History; MWU, Midwestern University; NMNH, Smithsonian National Museum of Natural History; OUVC, Ohio University Vertebrate Collections; UA, University of Arizona; UF, Florida Museum of Natural History; CA, cortical area; TA, total area; Imax, maximum second moment of inertia; Imin, minimum second moment of inertia. [file peerj-03-823-s002.docx]

Supplementary Table S2 **Additional geometric properties of humeral sections.**

| **Taxon** | **Specimen** | **CA (mm^2^)** | **TA (mm^2^)** | **I_max_ (mm^4^)** | **I_min_ (mm^4^)** |
| --- | --- | --- | --- | --- | --- |
| **Bats** |  |  |  |  |  |
| *Rhinolophus lepidus* | pers. coll. H1-2 | 0.419 | 0.887 | 0.049 | 0.042 |
| *Macrotus californicus* | UA 3767 H1-2 | 0.749 | 1.705 | 0.173 | 0.145 |
| *Myotis myotis* | Meier et al., 2013 | 0.828 | 1.650 | 0.179 | 0.149 |
| *Phyllostomus discolor* | UA 16197 H1-1 | 1.422 | 2.785 | 0.520 | 0.423 |
| *Noctilio leporinus* | UA 15743 H1-2 | 2.616 | 5.250 | 1.671 | 1.603 |
| *Rousettus leschenaultii* | pers. coll. H1-2 | 3.450 | 6.837 | 3.161 | 2.439 |
| *Pteropus vampyrus* | pers. coll. H1-2 | 14.554 | 29.974 | 59.189 | 45.477 |
|  |  |  |  |  |  |
| **Birds** |  |  |  |  |  |
| *Oceanodroma tethys* | NMNH 614194 | 1.462 | 2.185 | 0.411 | 0.278 |
| *Oceanites oceanicus* | CMNH 7752 | 1.681 | 2.783 | 0.651 | 0.415 |
| *Phalaenoptilus nuttallii* | MWU 264 H1-1 | 1.337 | 3.933 | 0.828 | 0.576 |
| *Bulweria bulweria* | NMNH 556263 | 4.532 | 5.515 | 2.695 | 2.024 |
| *Nothura darwinii* | UF 22260 H1-2 | 4.022 | 9.399 | 6.472 | 3.448 |
| *Crypturellus boucardi* | UF 44840 H1-1 | 5.848 | 18.333 | 19.909 | 10.294 |
| *Crypturellus cinnamomeus* | UA 8699 H2-1 | 7.254 | 18.066 | 21.677 | 12.827 |
| *Columba livia* | MWU 256 H1-2 | 6.337 | 20.084 | 21.894 | 13.245 |
| *Nothoprocta cinerascens* | UF 38951 H1-2 | 7.286 | 18.737 | 22.476 | 13.628 |
| *Calonectris diomedea* | OUVC 10438 | 17.752 | 28.157 | 61.753 | 47.707 |
| *Nothocercus nigrocapillus* | UF 43432 H1-2 | 6.797 | 19.386 | 23.788 | 12.504 |
| *Eudromia elegans* | UF 22257 H1-1 | 9.743 | 17.490 | 26.194 | 14.674 |
| *Tinamus major* | UF 44828 H1-2 | 13.730 | 39.143 | 101.268 | 49.223 |
| *Buteo jamaicensis* | OUVC 10506 | 19.666 | 53.125 | 156.644 | 117.224 |
| *Anhinga anhinga* | OUVC 10432 | 26.544 | 32.282 | 88.269 | 72.864 |
| *Phalacrocorax auritus* | OUVC 10482 | 24.786 | 38.211 | 112.971 | 90.972 |
| *Cathartes aura* | OUVC 9648 | 31.885 | 107.870 | 578.727 | 375.738 |
| *Pelecanus occidentalis* | OUVC 10484 | 32.200 | 169.577 | 920.348 | 663.401 |

Abbreviations: CMNH, Carnegie Museum of Natural History; MWU, Midwestern University; NMNH, Smithsonian National Museum of Natural History; OUVC, Ohio University Vertebrate Collections; UA, University of Arizona; UF, Florida Museum of Natural History; CA, cortical area; TA, total area; I_max_, maximum second moment of inertia; I_min_, minimum second moment of inertia.
