# Supplementary material for: Wing bone laminarity is not an adaptation for torsional resistance in bats
Source: PeerJ. 2015 Mar 5;3:e823. doi: 10.7717/peerj.823 (PMC4359045; doi:10.7717/peerj.823)
Supplement: Table S3 — A taxonomic name with an asterisk is spelled as it appears in mammalian (Bininda-Emonds et al., 2007) and avian (Jetz et al., 2012) tree files. Data were compiled from the following sources: (1) Kunz & Hood (2000); (2) Jin et al. (2012); (3) Zullinger et al. (1984); (4) McLean & Speakman (2000); (5) Kunz & Stern (1995); (6) Koehler & Barclay (2000); and (7) Starck & Ricklefs (1998). [file peerj-03-823-s003.docx]

Supplementary Table S3 **Compilation of maximum somatic growth rates.**

| **Taxon** | **Asymptotic mass (kg)** | **Mass at growth inflection (kg)** | **Max. growth rate (kg d^-1^)** | **Ref.** |
| --- | --- | --- | --- | --- |
| **Bats** |  |  |  |  |
| *Pipistrellus mimus* | 0.0034 | 0.0017 | 0.000094 | 1 |
| *Pipistrellus pipistrellus* | 0.0037 | 0.0019 | 0.000037 | 1 |
| *Pipistrellus subflavus* | 0.0052 | 0.0026 | 0.000169 | 1 |
| *Pipistrellus savii** | 0.0055 | 0.0028 | 0.000261 | 1 |
| *Rhinolophus marshalli* | 0.0064 | 0.0032 | 0.000176 | 2 |
| *Nycticeius humeralis* | 0.0064 | 0.0032 | 0.000096 | 1 |
| *Myotis lucifugus* | 0.0080 | 0.0029 | 0.000341 | 3 |
| *Plecotus auritus* | 0.0085 | 0.0025 | 0.000214 | 4 |
| *Tadarida brasiliensis* | 0.0106 | 0.0053 | 0.000451 | 1 |
| *Myotis velifer* | 0.0110 | 0.0040 | 0.000526 | 3 |
| *Molossus molossus* | 0.0123 | 0.0062 | 0.000246 | 1 |
| *Vespertilio superans* | 0.0153 | 0.0077 | 0.000421 | 1 |
| *Peropteryx kappleri* | 0.0154 | 0.0077 | 0.000308 | 1 |
| *Miniopterus schreibersi* | 0.0170 | 0.0063 | 0.000289 | 3 |
| *Eptesicus fuscus* | 0.0170 | 0.0063 | 0.000388 | 1 |
| *Carollia perspicillata* | 0.0172 | 0.0086 | 0.000172 | 1 |
| *Taphozous longimanus* | 0.0206 | 0.0103 | 0.000361 | 1 |
| *Antrozous pallidus* | 0.0227 | 0.0114 | 0.000542 | 5 |
| *Taphozous georgianus* | 0.0253 | 0.0127 | 0.000320 | 5 |
| *Noctilio albiventris* | 0.0254 | 0.0127 | 0.000381 | 1 |
| *Nyctalus lasiopterus* | 0.0262 | 0.0131 | 0.000786 | 1 |
| *Myotis myotis* | 0.0266 | 0.0133 | 0.000599 | 1 |
| *Lasiurus cinereus* | 0.0275 | 0.0138 | 0.000490 | 6 |
| *Nyctalus noctula* | 0.0276 | 0.0138 | 0.000828 | 1 |
| *Scotophilus heathi* | 0.0286 | 0.0143 | 0.000715 | 1 |
| *Eptesicus serotinus* | 0.0289 | 0.0145 | 0.000289 | 1 |
| *Desmodus rotundus* | 0.0299 | 0.0150 | 0.000075 | 1 |
| *Cynopterus brachyotis* | 0.0322 | 0.0161 | 0.000322 | 1 |
| *Cynopterus sphinx* | 0.0421 | 0.0211 | 0.000421 | 1 |
| *Phyllostomus discolor* | 0.0425 | 0.0213 | 0.000531 | 1 |
| *Artibeus jamaicensis* | 0.0528 | 0.0264 | 0.000528 | 1 |
| *Myotis daubentoni** | 0.0750 | 0.0375 | 0.001875 | 1 |
| *Phyllostomus hastatus* | 0.0779 | 0.0390 | 0.001169 | 1 |
| *Rousettus egyptiacus** | 0.1180 | 0.0434 | 0.000647 | 3 |
| *Pteropus pumilus* | 0.1718 | 0.0859 | 0.000859 | 1 |
| *Pteropus rodricensis* | 0.2485 | 0.1243 | 0.000621 | 1 |
| *Pteropus scapulatus* | 0.3791 | 0.1896 | 0.001896 | 1 |
| *Pteropus hypomelanus* | 0.5152 | 0.2576 | 0.001288 | 1 |
| *Pteropus poliocephalus* | 0.5839 | 0.2920 | 0.002920 | 1 |
| *Pteropus vampyrus* | 0.7555 | 0.3778 | 0.003778 | 1 |
| **Taxon** | **Asymptotic mass (kg)** | **Mass at growth inflection (kg)** | **Max. growth rate (kg d^-1^)** | **Ref.** |
| **Birds** |  |  |  |  |
| *Selasphorus rufus* | 0.0038 | 0.0019 | 0.000337 | 7 |
| *Regulus regulus* | 0.0055 | 0.0028 | 0.000665 | 7 |
| *Collocalia esculenta* | 0.0068 | 0.0034 | 0.000587 | 7 |
| *Lampornis clemenciae* | 0.0079 | 0.0040 | 0.000833 | 7 |
| *Troglodytes troglodytes* | 0.0089 | 0.0045 | 0.001086 | 7 |
| *Parus caeruleus* | 0.0096 | 0.0048 | 0.001202 | 7 |
| *Troglodytes aedon* | 0.0102 | 0.0051 | 0.001299 | 7 |
| *Parus atricapillus** | 0.0103 | 0.0052 | 0.001380 | 7 |
| *Geothlypis trichas* | 0.0106 | 0.0053 | 0.001575 | 7 |
| *Phylloscopus trochilus* | 0.0107 | 0.0054 | 0.001373 | 7 |
| *Spizella passerina* | 0.0119 | 0.0060 | 0.001474 | 7 |
| *Dendroica petechia* | 0.0122 | 0.0061 | 0.002540 | 7 |
| *Phoenicurus phoenicurus* | 0.0130 | 0.0065 | 0.002133 | 7 |
| *Saxicola rubetra* | 0.0143 | 0.0072 | 0.002212 | 7 |
| *Vireo olivaceus* | 0.0162 | 0.0081 | 0.002010 | 7 |
| *Tachycineta bicolor* | 0.0164 | 0.0082 | 0.002706 | 7 |
| *Erithacus rubecula* | 0.0176 | 0.0088 | 0.002040 | 7 |
| *Hirundo rustica* | 0.0184 | 0.0092 | 0.002343 | 7 |
| *Seiurus aurocapilla* | 0.0190 | 0.0095 | 0.002112 | 7 |
| *Zonotrichia albicollis* | 0.0202 | 0.0101 | 0.002863 | 7 |
| *Carpodacus mexicanus* | 0.0204 | 0.0102 | 0.002154 | 7 |
| *Sylvia atricapilla* | 0.0219 | 0.0110 | 0.001388 | 7 |
| *Passer domesticus* | 0.0230 | 0.0115 | 0.002638 | 7 |
| *Sylvia borin* | 0.0248 | 0.0124 | 0.001279 | 7 |
| *Oenanthe oenanthe* | 0.0250 | 0.0125 | 0.002799 | 7 |
| *Eremophila alpestris* | 0.0260 | 0.0130 | 0.004616 | 7 |
| *Zonotrichia leucophrys* | 0.0261 | 0.0131 | 0.002624 | 7 |
| *Lanius collurio* | 0.0270 | 0.0135 | 0.003018 | 7 |
| *Sialia mexicana* | 0.0275 | 0.0138 | 0.002880 | 7 |
| *Alauda arvensis* | 0.0317 | 0.0159 | 0.003630 | 7 |
| *Molothrus ater* | 0.0337 | 0.0169 | 0.004320 | 7 |
| *Tyrannus tyrannus* | 0.0357 | 0.0179 | 0.003596 | 7 |
| *Cardinalis cardinalis* | 0.0410 | 0.0205 | 0.004128 | 7 |
| *Plectrophenax nivalis* | 0.0418 | 0.0209 | 0.004464 | 7 |
| *Agelaius phoeniceus* | 0.0431 | 0.0216 | 0.004734 | 7 |
| *Pipilo aberti* | 0.0468 | 0.0234 | 0.003903 | 7 |
| *Charadrius hiaticula* | 0.0480 | 0.0240 | 0.002662 | 7 |
| *Lanius ludovicianus* | 0.0486 | 0.0243 | 0.005400 | 7 |
| *Turdus migratorius* | 0.0624 | 0.0312 | 0.007560 | 7 |
| *Sturnus vulgaris* | 0.0750 | 0.0375 | 0.007251 | 7 |
| *Falco sparverius* | 0.1160 | 0.0580 | 0.006195 | 7 |
| *Nucifraga caryocatactes* | 0.1470 | 0.0735 | 0.011690 | 7 |
| **Taxon** | **Asymptotic mass (kg)** | **Mass at growth inflection (kg)** | **Max. growth rate (kg d^-1^)** | **Ref.** |
| **Birds (continued)** |  |  |  |  |
| *Tringa totanus* | 0.1500 | 0.0750 | 0.003528 | 7 |
| *Pica pica* | 0.1589 | 0.0795 | 0.013870 | 7 |
| *Asio otus* | 0.2520 | 0.1260 | 0.015810 | 7 |
| *Columba livia* | 0.3680 | 0.1840 | 0.023386 | 7 |
| *Tyto alba* | 0.5332 | 0.2666 | 0.024147 | 7 |
| *Alca torda* | 0.5890 | 0.2945 | 0.012700 | 7 |
| *Corvus cryptoleucus* | 0.6400 | 0.3200 | 0.028350 | 7 |
| *Lagopus lagopus* | 0.6580 | 0.3290 | 0.015543 | 7 |
| *Anas platyrhynchos* | 1.0200 | 0.5100 | 0.019921 | 7 |
| *Buteo jamaicensis* | 1.4750 | 0.7375 | 0.047840 | 7 |
| *Bubo scandiaca** | 2.0260 | 1.0130 | 0.063165 | 7 |
| *Phalacrocorax carbo* | 2.1000 | 1.0500 | 0.071736 | 7 |
| *Gallus gallus* | 2.7100 | 1.3550 | 0.026929 | 7 |
| *Grus canadensis* | 3.8900 | 1.9450 | 0.049948 | 7 |

A taxonomic name with an asterisk is spelled as it appears in mammalian (Bininda-Emonds et al., 2007) and avian (Jetz et al., 2012) tree files. Data were compiled from the following sources: (1) Kunz and Hood (2000); (2) Jin et al. (2012); (3) Zulllinger et al. (1984); (4) McLean and Speakman (2000); (5) Kunz and Stern (1995); (6) Koehler and Barclay (2000); and (7) Starck and Ricklefs (1998).
