# Supplementary material for: Wing bone laminarity is not an adaptation for torsional resistance in bats
Source: PeerJ. 2015 Mar 5;3:e823. doi: 10.7717/peerj.823 (PMC4359045; doi:10.7717/peerj.823)
Supplement: Table S4 — A taxonomic name with an asterisk is spelled as it appears in mammalian (Bininda-Emonds et al., 2007) and avian (Jetz et al., 2012) tree files. Data were compiled from the following sources: (1) Nagy, Girard & Brown (1999); (2) Voigt, Kelm & Visser (2006); (3) Geiser & Coburn (1999); (4) Hudson, Isaac & Reuman (2013); and (5) Delorme & Thomas (1999). [file peerj-03-823-s004.docx]

Supplementary Table S4 **Compilation of field metabolic rates.**

| **Taxon** | **Mass (kg)** | **Field metabolic rate (W)** | **Ref.** |
| --- | --- | --- | --- |
| **Bats** |  |  |  |
| *Pipistrellus pipistrellus* | 0.0073 | 29.3 | 1 |
| *Plecotus auritus* | 0.0085 | 27.6 | 1 |
| *Glossophaga commissarisi* | 0.0087 | 45.7 | 2 |
| *Myotis lucifugus* | 0.0090 | 29.9 | 1 |
| *Anoura caudifera* | 0.0115 | 51.9 | 1 |
| *Macrotus californicus* | 0.0130 | 21.5 | 1 |
| *Syconycteris australis* | 0.0174 | 76.9 | 3 |
| *Carollia brevicauda* | 0.0182 | 50.0 | 2 |
| *Carollia perspicillata* | 0.0190 | 97.2 | 4 |
| *Eptesicus fuscus* | 0.0208 | 43.6 | 1 |
| *Artibeus jamaicensis* | 0.0368 | 131.3 | 5 |
| *Phyllostomus hastatus* | 0.0808 | 146.0 | 1 |
| *Rousettus egyptiacus** | 0.1567 | 352.1 | 5 |
| *Pteropus poliocephalus* | 0.8500 | 590.4 | 5 |
| **Birds** |  |  |  |
| *Archilochus alexandri* | 0.0037 | 29.1 | 1 |
| *Calypte anna* | 0.0045 | 31.8 | 1 |
| *Thalurania colombica* | 0.0049 | 37.9 | 1 |
| *Auriparus flaviceps* | 0.0066 | 30.0 | 1 |
| *Chalybura urochrysia* | 0.0072 | 57.9 | 1 |
| *Malurus cyaneus* | 0.0083 | 34.2 | 1 |
| *Lampornis clemenciae* | 0.0088 | 81.7 | 1 |
| *Parus ater** | 0.0095 | 47.4 | 1 |
| *Zosterops lateralis* | 0.0095 | 41.7 | 1 |
| *Nectarinia violacea** | 0.0095 | 66.2 | 1 |
| *Acanthorhynchus tenuirostris* | 0.0097 | 53.0 | 1 |
| *Troglodytes aedon* | 0.0106 | 60.8 | 1 |
| *Parus cristatus** | 0.0111 | 40.6 | 1 |
| *Parus montanus** | 0.0114 | 44.1 | 1 |
| *Parus caeruleus** | 0.0115 | 64.0 | 1 |
| *Eremiornis carteri** | 0.0120 | 51.5 | 1 |
| *Parus cinctus** | 0.0128 | 51.4 | 1 |
| *Ficedula hypoleuca* | 0.0135 | 65.8 | 1 |
| *Riparia riparia* | 0.0143 | 81.7 | 1 |
| *Hirundo tahitica* | 0.0144 | 64.9 | 1 |
| *Muscicapa striata* | 0.0144 | 52.0 | 1 |
| *Phylidonyris pyrrhopterus* | 0.0146 | 75.9 | 1 |
| *Ficedula albicollis* | 0.0159 | 78.6 | 1 |
| *Phylidonyris novaehollandiae* | 0.0173 | 77.6 | 1 |
| *Parus major* | 0.0180 | 97.4 | 1 |
| *Erithacus rubecula* | 0.0187 | 71.3 | 1 |
| **Taxon** | **Mass (kg)** | **Field metabolic rate (W)** | **Ref.** |
| **Birds (continued)** |  |  |  |
| *Passerculus sandwichensis* | 0.0187 | 80.4 | 1 |
| *Delichon urbicum* | 0.0190 | 79.8 | 1 |
| *Junco phaeonotus* | 0.0195 | 73.8 | 1 |
| *Junco hyemalis* | 0.0196 | 76.6 | 1 |
| *Hirundo rustica* | 0.0204 | 95.8 | 1 |
| *Prunella modularis* | 0.0212 | 86.0 | 1 |
| *Phainopepla nitens* | 0.0227 | 79.1 | 1 |
| *Cormobates leucophaea* | 0.0237 | 81.4 | 1 |
| *Oenanthe oenanthe* | 0.0243 | 91.4 | 1 |
| *Pyrrhula pyrrhula* | 0.0251 | 88.0 | 1 |
| *Philetairus socius* | 0.0255 | 48.7 | 1 |
| *Sialia mexicana* | 0.0274 | 95.0 | 1 |
| *Melopsittacus undulatus* | 0.0279 | 59.1 | 1 |
| *Certhilauda erythrochlamys* | 0.0285 | 64.3 | 1 |
| *Merops viridis* | 0.0343 | 85.3 | 1 |
| *Oceanites oceanicus* | 0.0423 | 119.0 | 1 |
| *Oceanodroma leucorhoa* | 0.0459 | 118.0 | 1 |
| *Mimus polyglottos* | 0.0476 | 121.0 | 1 |
| *Progne subis* | 0.0490 | 163.0 | 1 |
| *Actitis hypoleucos* | 0.0516 | 146.0 | 1 |
| *Calidris alba* | 0.0520 | 141.0 | 1 |
| *Neophema petrophila* | 0.0628 | 106.0 | 1 |
| *Cinclus cinclus* | 0.0637 | 196.0 | 1 |
| *Charadrius hiaticula* | 0.0748 | 302.0 | 1 |
| *Ceryle rudis* | 0.0760 | 210.0 | 1 |
| *Sturnus vulgaris* | 0.0787 | 269.0 | 1 |
| *Aethia pusilla* | 0.0803 | 350.0 | 1 |
| *Melanerpes formicivorus* | 0.0820 | 195.0 | 1 |
| *Geophaps plumifera* | 0.0870 | 76.0 | 1 |
| *Turdus merula* | 0.0960 | 179.0 | 1 |
| *Sterna paradisaea* | 0.1010 | 335.0 | 1 |
| *Arenaria interpres* | 0.1080 | 352.0 | 1 |
| *Pelecanoides georgicus* | 0.1090 | 464.0 | 1 |
| *Sterna hirundo* | 0.1270 | 343.0 | 1 |
| *Pelecanoides urinatrix* | 0.1370 | 557.0 | 1 |
| *Callipepla gambelii* | 0.1450 | 90.8 | 1 |
| *Barnardius zonarius* | 0.1450 | 189.0 | 1 |
| *Pachyptila desolata* | 0.1490 | 391.0 | 1 |
| *Alle alle* | 0.1640 | 696.0 | 1 |
| *Ptychoramphus aleuticus* | 0.1740 | 413.0 | 1 |
| *Sterna fuscata* | 0.1870 | 241.0 | 1 |
| *Ammoperdix heyi* | 0.1900 | 148.0 | 1 |
| *Anous stolidus* | 0.1950 | 352.0 | 1 |
| **Taxon** | **Mass (kg)** | **Field metabolic rate (W)** | **Ref.** |
| **Birds (continued)** |  |  |  |
| *Falco tinnunculus* | 0.2110 | 341.0 | 1 |
| *Cacatua roseicapilla** | 0.3070 | 349.0 | 1 |
| *Phaethon lepturus* | 0.3700 | 777.0 | 1 |
| *Cepphus grylle* | 0.3800 | 860.0 | 1 |
| *Puffinus pacificus* | 0.3840 | 614.0 | 1 |
| *Rissa tridactyla* | 0.3860 | 795.0 | 1 |
| *Alectoris chukar* | 0.3950 | 260.0 | 1 |
| *Uria lomvia* | 0.8340 | 1480.0 | 1 |
| *Uria aalge* | 0.9400 | 1870.0 | 1 |
| *Sula sula* | 1.0700 | 1220.0 | 1 |
| *Centrocercus urophasianus* | 2.5000 | 1540.0 | 1 |
| *Morus capensis* | 2.5800 | 3380.0 | 1 |
| *Phoebastria immutabilis* | 3.0700 | 1330.0 | 1 |
| *Morus bassanus* | 3.2100 | 4870.0 | 1 |
| *Thalassarche chrysostoma* | 3.7100 | 2390.0 | 1 |
| *Macronectes giganteus* | 3.8900 | 433.0 | 1 |
| *Diomedea exulans* | 8.4200 | 3350.0 | 1 |

A taxonomic name with an asterisk is spelled as it appears in mammalian (Bininda-Emonds et al., 2007) and avian (Jetz et al., 2012) tree files. Data were compiled from the following sources: (1) Nagy et al.(1999); (2) Voigt et al. (2006); (3) Geiser and Coburn (1999); (4) Hudson et al.(2013); and (5) Delorme and Thomas (1999).
