# Supplementary material for: Wing bone laminarity is not an adaptation for torsional resistance in bats
Source: PeerJ. 2015 Mar 5;3:e823. doi: 10.7717/peerj.823 (PMC4359045; doi:10.7717/peerj.823)
Supplement: Figure S1 — Circularly polarized light in cleared sections of standardized thickness reveals lamellar and parallel-fibered bone. Black to white intensity of polarized light corresponds to longitudinally oriented and transversely oriented collagen fibers, respectively. Representative views are from the lateral octant of (A) Rhinolophus lepidus, (B) Macrotus californicus, (C) Phyllostomus discolor, (D) Noctilio leporinus, (E) Rousettus leschenaultii, and (F) Pteropus vampyrus. Periosteal surface points up in each panel. Scale bar equals (A & B) 200 µm, (C) 300 µm, (D) 400 µm, (E) 480 µm, and (F) 1,200 µm. Digital slides are available at http://paleohistology.appspot.com. [file peerj-03-823-s005.pdf]

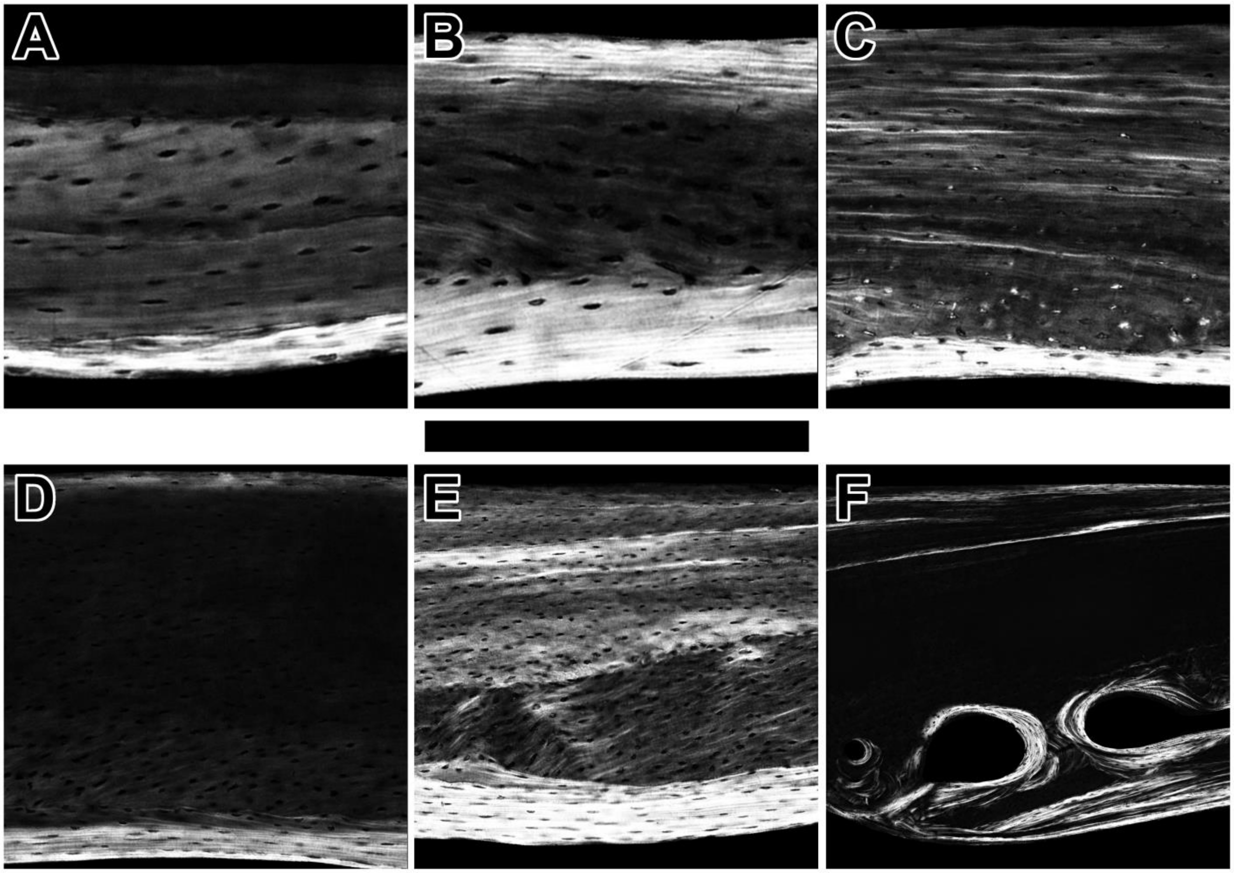


Figure S1  **Collagen fiber orientation of humeri in sampled bats.**  Circularly polarized light in cleared sections of standardized thickness reveals lamellar and parallel-fibered bone.  Black to white intensity of polarized light corresponds to longitudinally oriented and transversely oriented collagen fibers, respectively.  Representative views are from the lateral octant of (A) *Rhinolophus lepidus*, (B) *Macrotus californicus*, (C) *Phyllostomus discolor*, (D) *Noctilio leporinus*, (E) *Rousettus leschenaultii*, and (F) *Pteropus vampyrus*.  Periosteal surface points up in each panel. Scale bar equals (A & B) 200 µm, (C) 300 µm, (D) 400 µm, (E) 480 µm, and (F) 1200 µm.  Digital slides are available at http://paleohistology.appspot.com.
