# Supplementary material for: Wing bone laminarity is not an adaptation for torsional resistance in bats
Source: PeerJ. 2015 Mar 5;3:e823. doi: 10.7717/peerj.823 (PMC4359045; doi:10.7717/peerj.823)
Supplement: Figure S2 — Representative views are from the dorsal octant of (A) Phalaenoptilus nuttallii, (B) Nothura darwinii, (C) Crypturellus boucardi, (D) Crypturellus cinnamomeus, (E) Columba livia, (F) Nothoprocta cinerascens, (G) Nothocercus nigrocapillus, (H) Eudromia elegans, and (I) Tinamus major. Periosteal surface points up in each panel. Scale bar equals (A) 300 µm, (B & E) 480 µm, (C, D, F & G) 600 µm, and (H & I) 800 µm. Digital slides are available at http://paleohistology.appspot.com. [file peerj-03-823-s006.pdf]

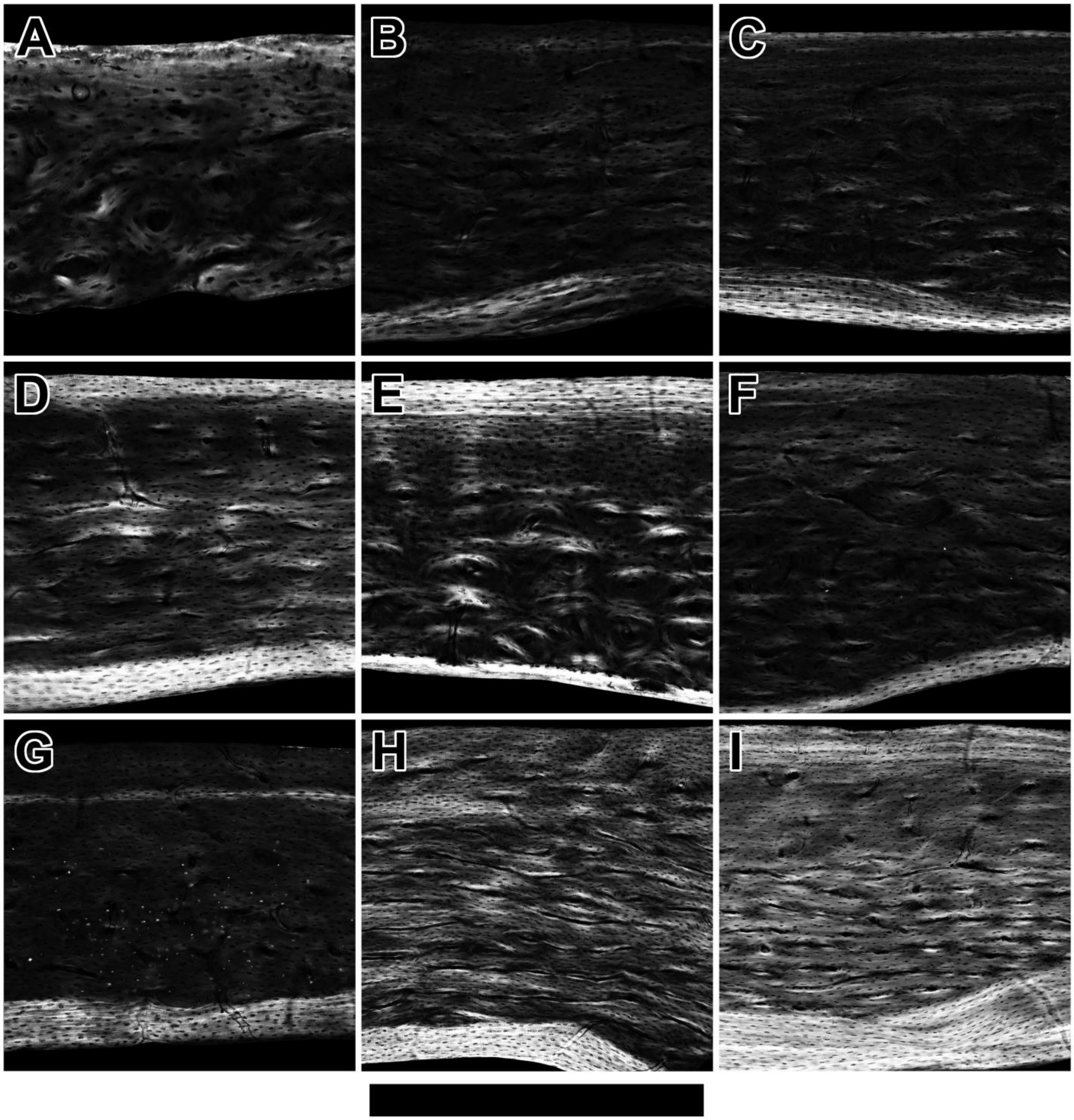

Figure S2 **Collagen fiber orientation of humeri in sampled birds.** Representative views are from the dorsal octant of (A) *Phalaenoptilus nuttallii*, (B) *Nothura darwinii*, (C) *Crypturellus boucardi*, (D) *Crypturellus cinnamomeus*, (E) *Columba livia*, (F) *Nothoprocta cinerascens*, (G) *Nothocercus nigrocapillus*, (H) *Eudromia elegans*, and (I) *Tinamus major*. Periosteal surface points up in each panel. Scale bar equals (A) 300 µm, (B & E) 480 µm, (C, D, F & G) 600 µm, and (H & I) 800 µm. Digital slides are available at http://paleohistology.appspot.com.
