# Supplementary material for: Wing bone laminarity is not an adaptation for torsional resistance in bats
Source: PeerJ. 2015 Mar 5;3:e823. doi: 10.7717/peerj.823 (PMC4359045; doi:10.7717/peerj.823)
Supplement: Figure S3 — Sections stained with toluidine blue reveal lamellar bone, a complex canalicular network, resorption fronts, and canals of secondary osteons, which were excluded from analysis. Representative views are from the posterior octant of (A) Rhinolophus lepidus, (B) Macrotus californicus, (C) Phyllostomus discolor, (D) Noctilio leporinus, (E) Rousettus leschenaultii, and (F) Pteropus vampyrus. Periosteal surface points up in each panel. Scale bar equals (A) 150 µm, (B) 200 µm, (C & D) 300 µm, (E) 480 µm, and (F) 800 µm. Digital slides are available at http://paleohistology.appspot.com. [file peerj-03-823-s007.pdf]

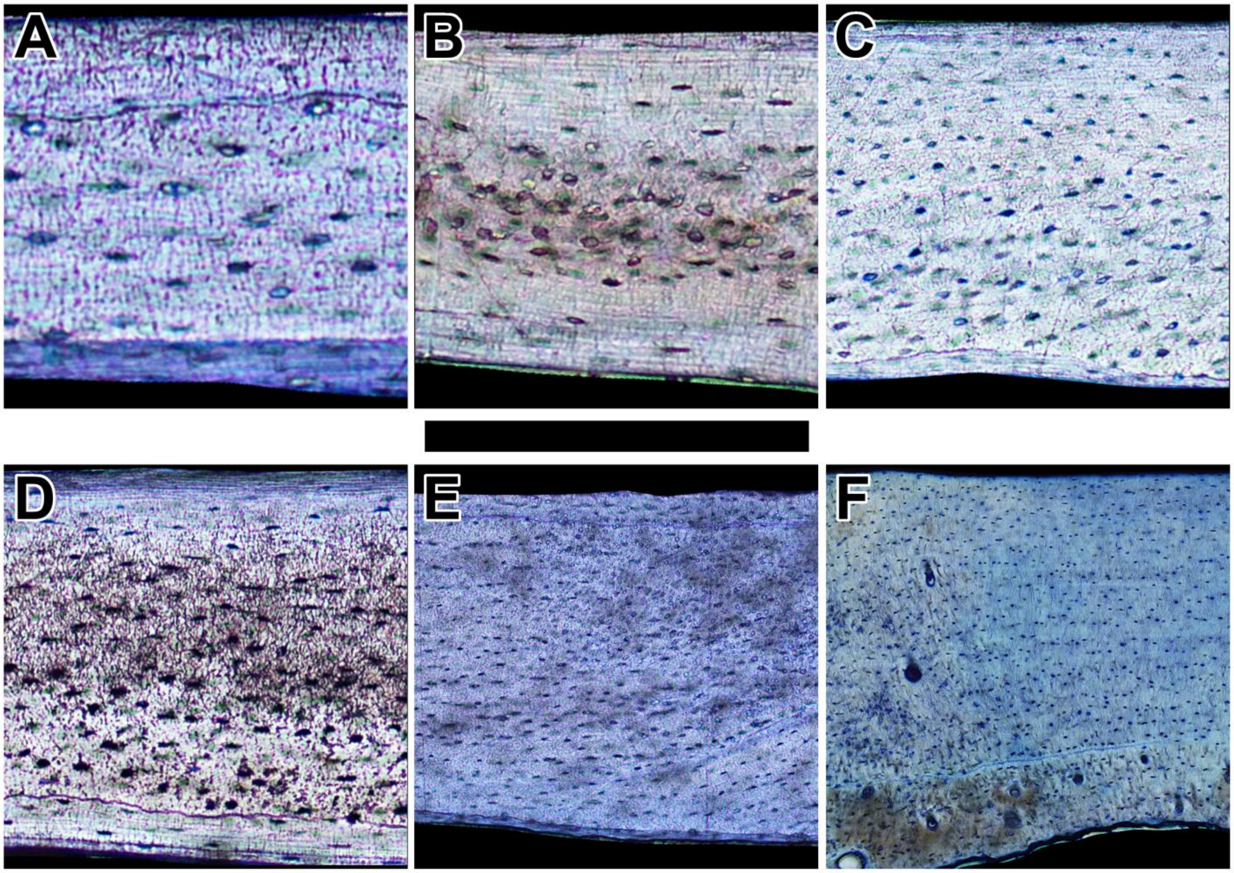

Figure S3 **Bone histology of humeri in sampled bats stained to highlight cement lines.** Sections stained with toluidine blue reveal lamellar bone, a complex canalicular network, resorption fronts, and canals of secondary osteons, which were excluded from analysis. Representative views are from the posterior octant of (A) *Rhinolophus lepidus*, (B) *Macrotus californicus*, (C) *Phyllostomus discolor*, (D) *Noctilio leporinus*, (E) *Rousettus leschenaultii*, and (F) *Pteropus vampyrus*. Periosteal surface points up in each panel. Scale bar equals (A) 150 µm, (B) 200 µm, (C & D) 300 µm, (E) 480 µm, and (F) 800 µm. Digital slides are available at http://paleohistology.appspot.com.
