# Supplementary material for: Wing bone laminarity is not an adaptation for torsional resistance in bats
Source: PeerJ. 2015 Mar 5;3:e823. doi: 10.7717/peerj.823 (PMC4359045; doi:10.7717/peerj.823)
Supplement: Figure S4 — Sections stained with toluidine blue reveal arrest lines and canals of secondary osteons, which were excluded from analysis. Representative views are from the caudal octant of (A) Phalaenoptilus nuttallii, (B) Nothura darwinii, (C) Crypturellus boucardi, (D) Crypturellus cinnamomeus, (E) Columba livia, (F) Nothoprocta cinerascens, (G) Nothocercus nigrocapillus, (H) Eudromia elegans, and (I) Tinamus major. Periosteal surface points up in each panel. Scale bar equals (A) 200 µm, (B & G) 480 µm, (C, D, F, & I) 600 µm, (E) 400 µm, and (H) 800 µm. Digital slides are available at http://paleohistology.appspot.com. [file peerj-03-823-s008.pdf]

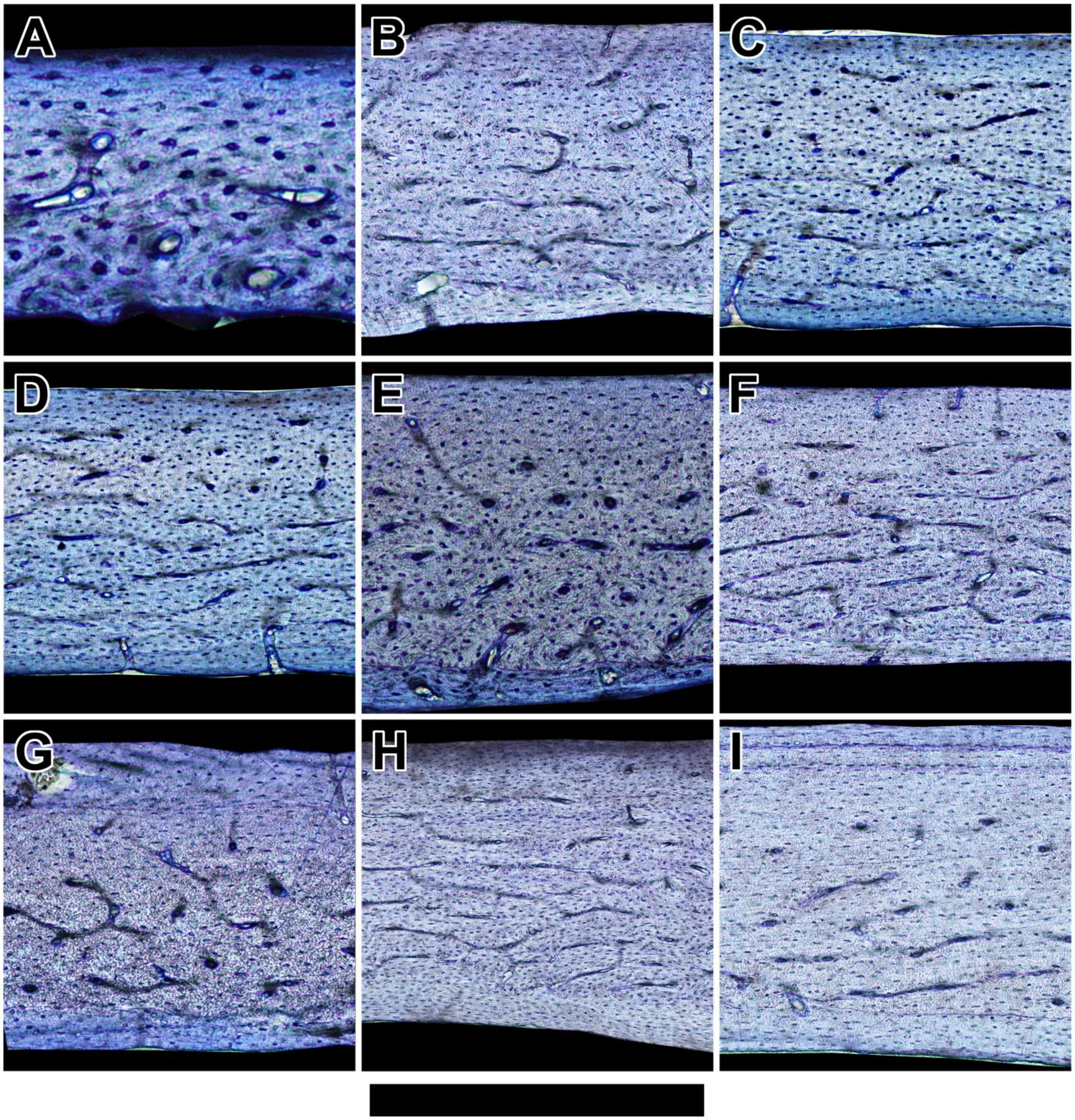

Figure S4 **Bone histology of humeri in sampled birds stained to highlight cement lines.** Sections stained with toluidine blue reveal arrest lines and canals of secondary osteons, which were excluded from analysis. Representative views are from the caudal octant of (A) *Phalaenoptilus nuttallii*, (B) *Nothura darwinii*, (C) *Crypturellus boucardi*, (D) *Crypturellus cinnamomeus*, (E) *Columba livia*, (F) *Nothoprocta cinerascens*, (G) *Nothocercus nigrocapillus*, (H) *Eudromia elegans*, and (I) *Tinamus major*. Periosteal surface points up in each panel. Scale bar equals (A) 200 μm, (B & G) 480 μm, (C, D, F, & I) 600 μm, (E) 400 μm, and (H) 800 μm. Digital slides are available at http://paleohistology.appspot.com.
